# Large-Scale Screen for Modifiers of Ataxin-3-Derived Polyglutamine-Induced Toxicity in *Drosophila*

**DOI:** 10.1371/journal.pone.0047452

**Published:** 2012-11-05

**Authors:** Hannes Voßfeldt, Malte Butzlaff, Katja Prüßing, Róisín-Ana Ní Chárthaigh, Peter Karsten, Anne Lankes, Sabine Hamm, Mikael Simons, Boris Adryan, Jörg B. Schulz, Aaron Voigt

**Affiliations:** 1 Department of Neurology, University Medical Center, RWTH Aachen, Aachen, Germany; 2 Max Planck Institute for Experimental Medicine, Göttingen, Germany; 3 Department of Neurology, University of Göttingen, Göttingen, Germany; 4 Cambridge Systems Biology Centre, University of Cambridge, Cambridge, United Kingdom; 5 Jülich-Aachen Research Alliance (JARA) Brain – Translational Brain Medicine, Aachen, Germany; Brigham and Women's Hospital, Harvard Medical School, United States of America

## Abstract

Polyglutamine (polyQ) diseases represent a neuropathologically heterogeneous group of disorders. The common theme of these disorders is an elongated polyQ tract in otherwise unrelated proteins. So far, only symptomatic treatment can be applied to patients suffering from polyQ diseases. Despite extensive research, the molecular mechanisms underlying polyQ-induced toxicity are largely unknown. To gain insight into polyQ pathology, we performed a large-scale RNAi screen in *Drosophila* to identify modifiers of toxicity induced by expression of truncated Ataxin-3 containing a disease-causing polyQ expansion. We identified various unknown modifiers of polyQ toxicity. Large-scale analysis indicated a dissociation of polyQ aggregation and toxicity.

## Introduction

The group of polyglutamine (polyQ) diseases comprises nine dominant heritable neurodegenerative disorders, including Huntington's disease, spinobulbar muscular atrophy and several spinocerebellar ataxias (SCA). All nine disorders are caused by gain-of-function mutations, resulting in an expanded trinucleotide (CAG) repeat tract, translated into a polyQ expansion in the respective disease protein. Spinocerebellar ataxia type 3 (SCA3) or Machado-Joseph disease is the most frequent among the SCA subtypes, comprising about 21% of the worldwide cases of autosomal dominant cerebellar ataxias [1]. In SCA3, the disease protein Ataxin-3 harbors an abnormally elongated polyQ expansion, causative for disease [2]. Such elongated polyQ expansions are the common theme in various other disorders, the reason why these disorders are often summarized as polyQ diseases. The disease-linked proteins share no homology to each other apart from the polyQ tract, suggesting a common pathogenic mechanism leading to the development of disease. According to the toxic fragment hypothesis, the polyQ tract itself is the actual toxic species due to its ability to cause neurodegeneration [3], [4], [5]. There is an inverse correlation between repeat number and age of onset. Additionally, severity of the disease increases with the length of the CAG tract [6], [7]. Expansion of the polyQ stretch in the disease protein renders the mutant variant prone to aggregation [8]. The actual inclusions are formed through putative toxic intermediates [9]. Nevertheless, the toxicity of the different aggregating species is still under discussion, favoring oligomers of the disease proteins as the trigger of neuronal dysfunction and eventually neurodegeneration [10]. Additionally, nuclear translocation of proteolytically cleaved polyQ proteins and formation of nuclear inclusions are early events in pathogenesis and known to be hallmarks in polyQ diseases [11], [12].

Impairment of the ubiquitin-proteasomal system (UPS) seems to be a key factor in polyQ pathogenesis [13]. UPS activity is needed to clear aggregates of mutated proteins. Cells with impaired UPS therefore fail to attenuate the toxic effects of polyQ species [14].

Besides misfolding of the mutant proteins and impaired cellular protein homeostasis, many other hypotheses have been proposed to explain polyQ disease pathogenesis. Among these are deleterious protein interactions, transcriptional dysregulation, mitochondrial dysfunction, impaired axonal transport, anomalous neuronal signaling and RNA toxicity [15], [16], [17].

With regard to similar toxicity of heterogeneous proteins in different cellular and spatial settings, there is overwhelming need for insight into polyQ protein-interacting genes in order to decipher the processes involved in neurotoxicity. *Drosophila* has proven to be a valuable model organism in research of neurodegenerative diseases, not least in diverse screening approaches [18], [19], [20], [21]. Changes in the polyQ-induced rough eye phenotype (REP) are easily accessible and thus an ideal tool to perform high-throughput screening for genetic modifiers of polyQ toxicity. Utilizing an RNAi library comprised of almost all fly genes having a human ortholog [22], we conducted a *Drosophila* screen set to identify genetic interactors of polyQ toxicity. Computational analysis helped to reveal common pathways of the discovered modifier genes, providing insights into possible disease mechanisms leading to neurodegeneration in polyQ disorders.

## Results

### Identification of novel modifiers of polyQ toxicity

**Figure 1:**
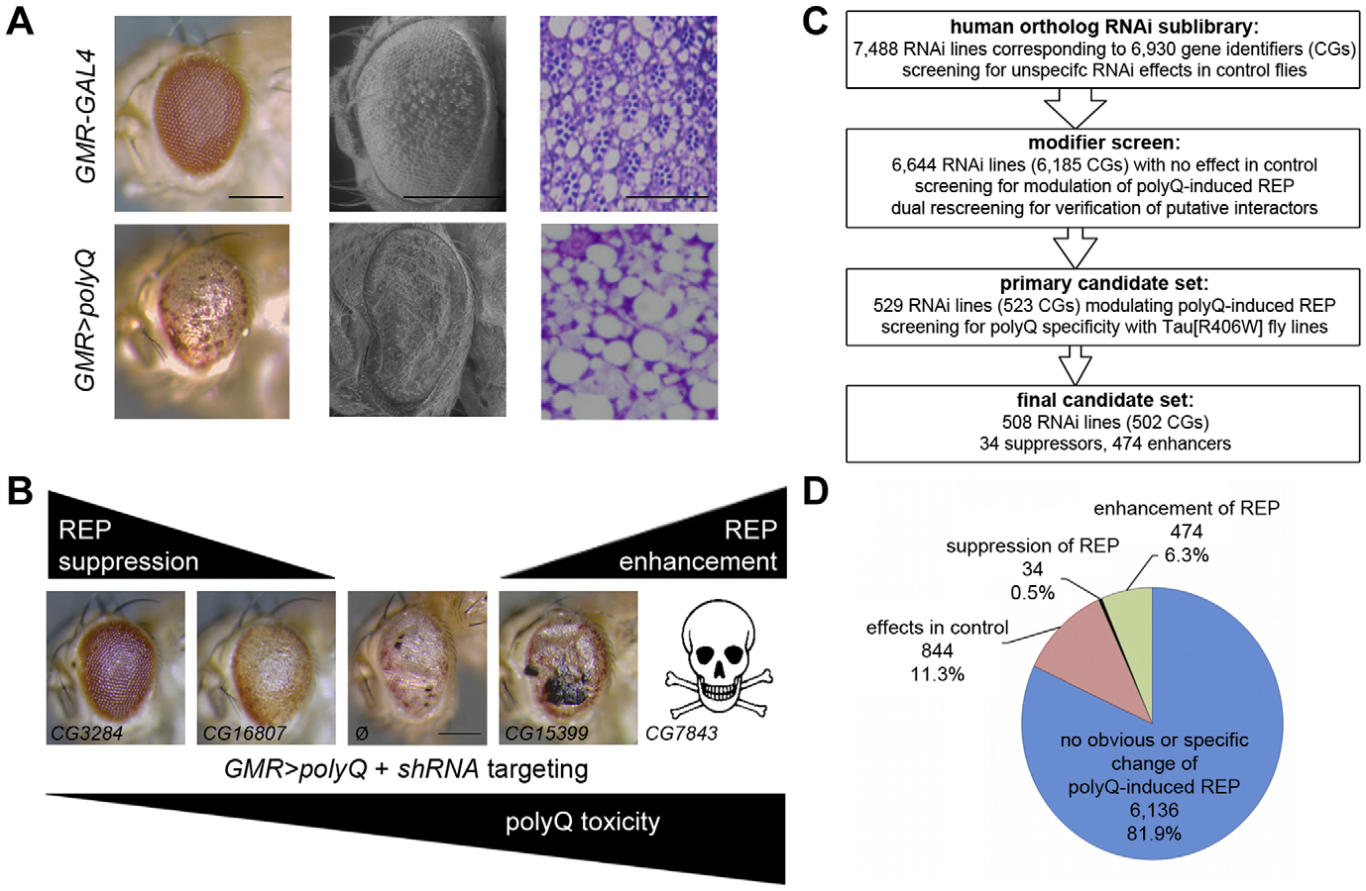
Screening for modifiers of polyQ-induced toxicity. (**A**) Rough eye phenotype (REP) used as a primary readout for screening. Compared to control (upper panels), eye-specific (*GMR-GAL4*) expression of polyQ (lower panels) induces disturbances of the external eye texture, e. g. depigmentation of the compound eye observed by light microscopy (left) and as depicted in scanning electron micrographs (middle). Toluidine blue-stained semi-thin eye sections reveal that the disturbance of external eye structures is accompanied by degeneration of retinal cells (right). (**B**) Modification of the polyQ-induced REP by enhancers and suppressors. VDRC transformants used to silence respective genes: *CG3284* (11219), *CG16807* (23843), *CG15399* (19450) and *CG7843* (22574). (**C**) Flow chart of the screening procedures to identify modifiers of polyQ-induced toxicity. (**D**) Brief summary of screen results. Scale bars represent either 200 µm in eye pictures or 50 µm in semi-thin eye sections.

Flies with stable expression of an Ataxin-3-derived polyQ tract (78 glutamines [23]) in all post-mitotic cells of the fly eye (*GMR>polyQ*) display a REP characterized by pigment loss, a disturbed external surface and appearance of necrotic spots. This easily visible REP is a consequence of degenerating photoreceptors and other retinal cells (Figure 1A). The severity of the REP has also been shown to be sensitive towards modifications by second-site mutations (Figure 1B) [18], [19], [20], [21]. To screen for modifiers of polyQ toxicity, we used a recently established *Drosophila* RNAi library (VDRC) [22]. This library is comprised of transgenes, expressing inverted repeat sequences forming short hairpin RNAs under UAS control. Via processing of these double stranded RNAs, small interfering RNAs are produced, which eventually leads to silencing of the targeted gene by RNA interference (RNAi). As we are interested in human disease, we restricted our analysis to all fly genes of which a human ortholog could be identified (6,930 genes, full list is available on request) comprising roughly 45% of all protein coding genes in the fly. First, we tested if RNAi-mediated silencing of a given gene caused any alteration of external eye structures. In case *GMR-GAL4*-driven RNAi induced changes in adult eyes, these lines were excluded from future analysis. For the actual screen, *GMR>polyQ* flies were crossed to the remaining RNAi lines. In the F1 generation, flies with combined eye-specific polyQ expression and RNAi-mediated gene silencing were analyzed for enhancement or suppression of the REP (Figure 1 B, C). Modifiers were considered as candidates if obvious changes on polyQ-induced REP were observed. Mild alterations of the REP appeared frequently and were categorized as subtle modification. An overview of all candidates is presented in Table S1. Given the large number of candidates, we were unable to prove effective silencing of gene expression by RNAi for all candidates. However, if a target gene was reported to be required for vitality, we tried to confirm the lethal phenotype by ubiquitous expression (*Act-GAL4*) of the respective RNAi transgene. Ubiquitous silencing of these genes caused almost invariably lethality (82% of genes analyzed), while silencing of the remaining genes at least resulted in semi-lethality or highly reduced offspring number (Table S1). Thus, we assume that the majority of the RNAi transgenes provide efficient silencing of their target.

### Modifiers are specific for polyQ-induced toxicity

In our primary screen, we identified a large number of enhancers and few suppressors of polyQ-induced toxicity (Figure 1D). Next, we analyzed if the identified modifiers are specific for polyQ-induced toxicity. Mutations in the *tau* gene like Tau[R406W] cause Frontotemporal Dementia and Parkinsonism linked to chromosome 17 (FTDP-17) [24]. *GMR*-driven expression of Tau (WT and FTLD-17-linked mutant variants) results in REPs, that are also sensitive towards genetic modifications. Such REPs induced by Tau variants (e. g. WT and V337M) have previously been used for modifier screens [25], [26], [27]. Using the Tau[R406W]-induced REP, we asked if identified polyQ modifiers might have similar effects on Tau-induced toxicity. Interestingly, only 4% of polyQ modifiers (21) similarly affected the Tau-dependent REP (Table 1). Silencing of these genes might affect the cell's folding environment and therefore have an impact on toxicity of the two aggregation-prone proteins polyQ and Tau[R406W], respectively. In case of suppressor activity on both REPs, gene silencing might influence expression strength of the toxic proteins (Tau[R406W] or polyQ) *per se*. We therefore considered these candidates as a separate group. The low number of candidates showing modification in both disease models implied that most of the identified modifiers are rather specific for polyQ-induced toxicity.

**Table 1:** List of unspecific modifiers of polyQ-induced toxicity.

Name/CG	Effect on Tau-induced REP	Effect on polyQ-induced REP	Predicted molecular function/biological process (as listed on flybase.org)
*Rab30/CG9100*	E	E	GTPase activity/involved in vesicle sorting and transport
*Aats-his/CG6335*	E	E	histidine-tRNA ligase/histidyl-tRNA aminoacylation
*MED14/CG12031*	E	E	protein binding/transcription from RNA polymerase II promoter
*Prp8/CG8877*	E	E	unknown/nuclear mRNA splicing, via spliceosome
*Nelf-E/CG5994*	E	E	mRNA binding/negative regulation of transcription from RNA polymerase II promoter during mitosis
*RpS10a/CG12275*	E	E	Structural constituent of ribosome/neurogenesis
*-/CG11985*	E	E	unknown/mitotic spindle organization
*Prosbeta2/CG3329*	E	E	endopeptidase activity/catalytic constituent of the proteasome (beta-subunit), protein degradation
*Rpn9/CG10230*	E	E	endopeptidase activity/regulation of exit from mitosis, protein degradation
*bic/CG3644*	E	E	unknown/regulation of establishment of protein localization, RNA binding, intracellular mRNA localization
*MRG15/CG6363*	S	S	unknown/chromatin silencing
*Hop/CG2720*	S	S	unfolded protein binding/protein folding
*-/CG6364*	E	E	Uridine kinase activity/phagocytosis, engulfment
*-/CG6873*	E	E	Actin binding, polymerization/neurogenesis
*Nrx-IV/CG6827*	E	E	transmembrane signaling receptor activity/dorsal closure; nerve maturation; regulation of tube size, open tracheal system; establishment of glial blood-brain barrier; septate junction assembly; axon ensheathment.
*CycJ/CG10308*	E	E	cyclin-dependent protein kinase regulator activity/mitotic cell cycle, embryonic; mitosis
*-/CG8086*	E	E	unknown/neurogenesis
*bru/CG2478*	E	E	unknown/cytokinesesis
*-/CG8108*	E	E	zinc ion binding/unknown
*vnc/CG11989*	E	E	peptide alpha-N-acetyltransferase activity/oogenesis, neurogenesis
*Smg5/CG8954*	E	E	unknown/nuclear-transcribed mRNA catabolic process, nonsense-mediated decay

Table lists gene name (if applicable) and gene ID of all candidates identified to have a similar effect on polyQ- and Tau-induced REPs. Mode of modification is indicated (enhancement (E), suppression (S)). A brief summary of the molecular and biological functions assigned to the identified gene products is listed.

### Suppression of polyQ-induced toxicity is not restricted to the retina

Our primary screen was based on retina degeneration. Consequently, identified modifications might be specific to the retina. We wanted to test whether our candidates also protect against polyQ-induced toxicity in neurons different from photoreceptors. Pan-neural (*elav-GAL4*) expression of the *polyQ* construct used for screening did not result in viable offspring [23]. However, in combination with identified suppressors, a large portion of tested suppressors rescued lethality in these flies (Table S2). Thus, protective effects on polyQ-induced toxicity of the majority of suppressors are not restricted to photoreceptors but also apply to other neuron types.

### Toxicity does not correlate with polyQ aggregation

**Figure 2:**
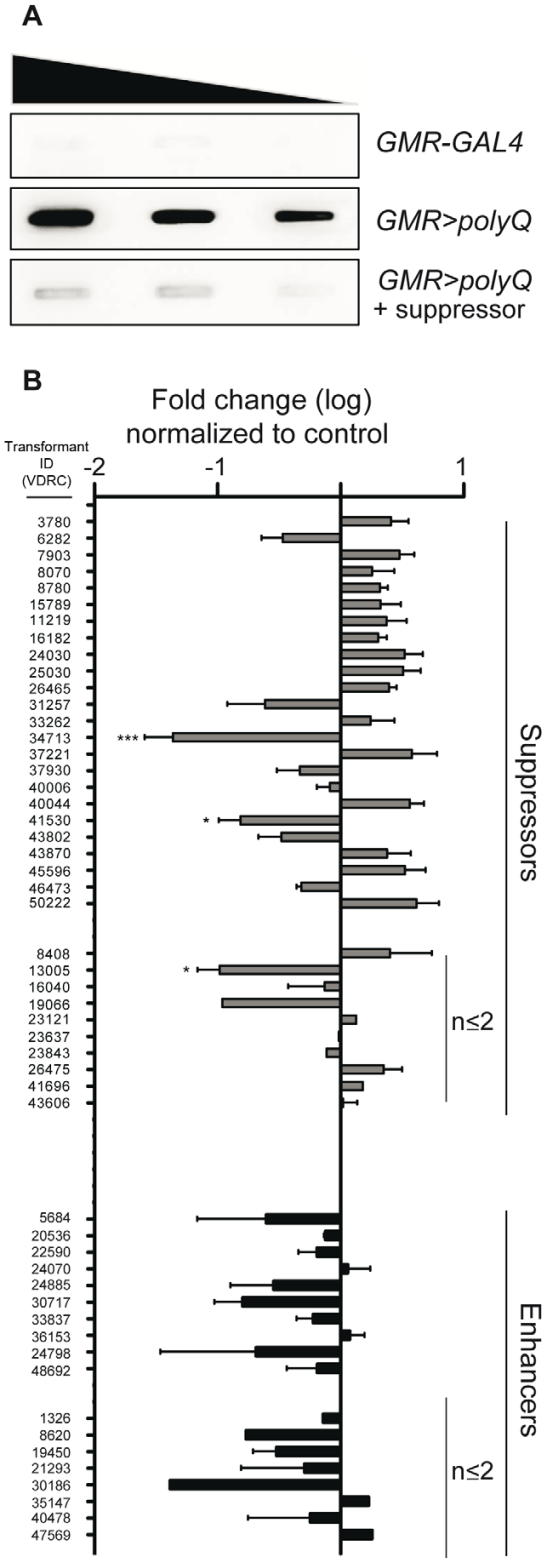
Analysis of polyQ aggregate load. (**A**) Exemplified filter retardation analysis to visualize polyQ aggregates. Decreasing amounts of loaded protein derived from fly heads of control (*GMR-GAL4*, top), *GMR>polyQ* (middle) or *GMR>polyQ* in combination with a candidate suppressor (bottom). (**B**) Densitometric measures of filter retardation analysis. Data depicted as fold change compared to control (*GMR>polyQ*) for suppressors and enhancers of polyQ-induced toxicity. Independent homogenates (if available) were used for repetitions. In case of none or only one independent repetition n≤2 is indicated. In all other cases, number of independent repetitions is n≥3. Significant changes are indicated * p<0.05; *** p<0.001.

Aggregation of proteins containing an elongated glutamine expansion is a common feature of polyQ diseases [12], [14], [28], [29], [30]. In addition, polyQ aggregation is considered to be at least partially causative for toxicity. Therefore we assumed that suppressors of polyQ toxicity identified in our screen might reduce polyQ aggregation, whereas enhancers might increase aggregate load. The so-called filter retardation assay is a widely used method to visualize SDS-insoluble, aggregated polyQ-containing proteins or peptides (Figure 2A) [31]. The main number of candidate enhancers (457) caused a lethal interaction in combination with polyQ expression. Thus, the absence of viable progeny did not allow to test for aggregation. Nevertheless, we analyzed remaining modifiers with respect to polyQ aggregation (Figure 2B, Figure S1). Only 3 of 34 suppressors analyzed showed a significant reduction of aggregate load. Despite that, the analyzed suppressors displayed no clear trend with respect to aggregate load. An increase as well as a decrease of aggregates was observed. In contrast, most of the analyzed enhancers of polyQ toxicity displayed a slight reduction in aggregate load. In summary, we can conclude that obvious changes in toxicity do not seem to coincide with equivalent changes in aggregate load.

### Computational analysis of candidates implies an involvement of multiple processes in polyQ toxicity

**Figure 3:**
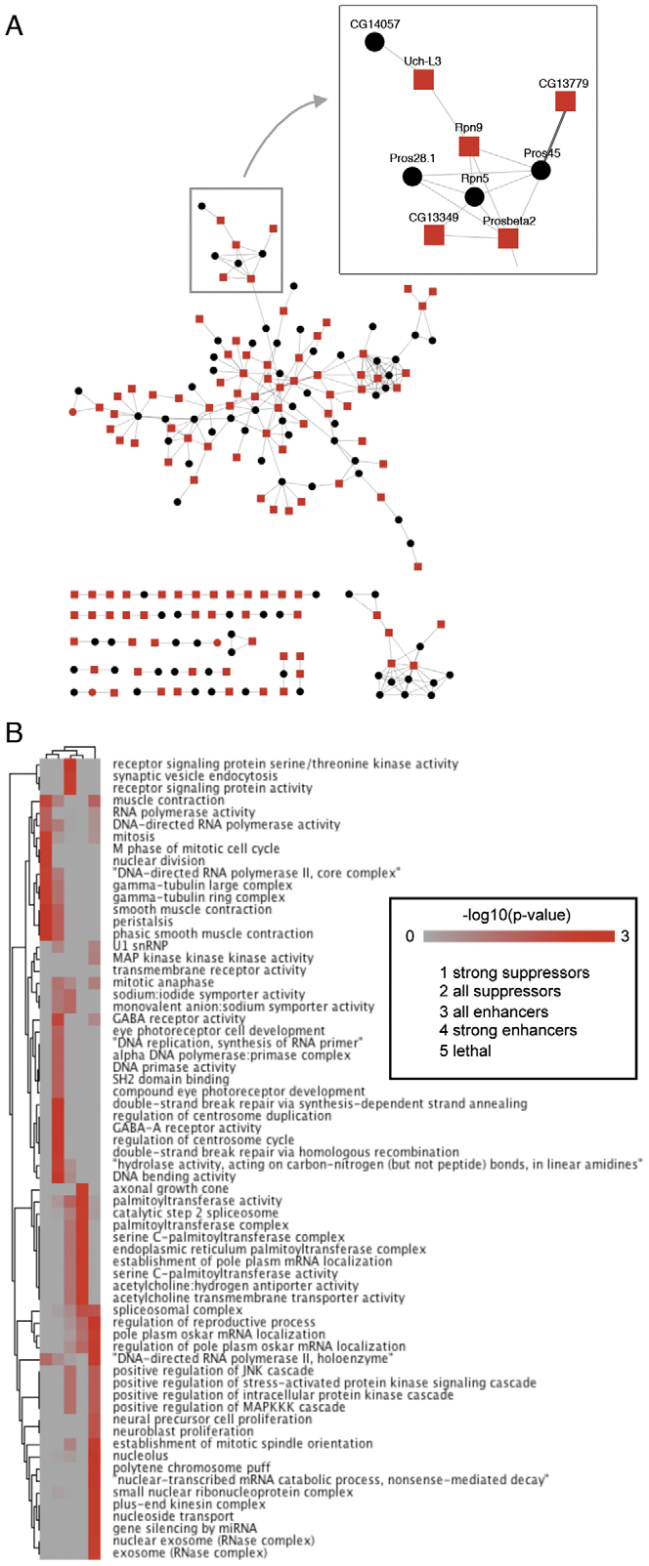
Computational analysis of modifiers of polyQ-induced toxicity. (**A**) Meta-interaction network displaying modifiers of polyQ toxicity. Only candidates causing a robust modification of the REP (red) as well as directly interacting subtle modifiers (black) were retained from an initial network of more than 5 k genes with 20 k interactions [32]. One local cluster of functionally interacting modifiers is highlighted. (**B**) Gene Ontology analysis of these candidate gene groups. Shown are -log_10_(p-value) scores for GO term enrichment for candidate gene groups (horizontal axis, see inset for group identities) and GO term (vertical). The matrix incorporates the structure of the GO hierarchy and is based on the Topology Weighted Term-algorithm as implemented in Ontologizer (terms with a p-value<0.005 are shown).

Finally, we performed a computational analysis to identify cellular processes/pathways, which might be involved in polyQ toxicity (Figure 3, Figure S2). We first overlaid our candidate genes onto the meta-interaction network from Costello and co-workers [32]. We were only interested in those network components that showed a high degree of clustering. To increase the number of candidate genes, we included subtle modifiers. Throughout the primary screen, we categorized suppressors of the polyQ-induced REP in following groups: (1) wildtype-like, (2) robust and (3) subtle suppression. Enhancers were categorized in: (5) subtle and (6) robust enhancement of REP, (7) indicating lethality. Only strong candidate genes (categories 1, 2, 6, 7) and subtle candidates (categories 3, 5) that are directly interacting with strong ones were retained in the network. The resulting network in Figure 3A consists of 195 genes and 277 interactions. Note that this network does not represent a cohesive functional module, but only serves to highlight interacting components with primarily similar functions. Importantly, this strategy re-discovered a set of proteasomal proteins (Figure 3A, inset) previously implicated in polyQ toxicity [33]. The final network graph is available for direct visualization in Cytoscape (Dataset S1, Cytoscape is available at http://www.cytoscape.org/download.php).

Assuming that distinct Gene Ontology (GO) functional categories could be enriched in our different candidate groups, we treated suppressors (strong/weak), enhancers (strong/weak) and lethal candidates separately in the analysis of over-represented terms (Figure 3B). Interestingly, this shows mostly separated functional categories for the different candidate groups, with some shared functionality between strong and weak representatives of enhancers or suppressors, respectively. We therefore also generated candidate gene lists based on combinations of candidate groups and tested them for enrichment, using either their explicit GO annotation (Figure S2, upper panel) or inferred functionality (Topology Weighted-annotation considering the hierarchy of the ontology, Figure S2, lower panel) (raw data available in Dataset S1, the visualization tool Genesis is available at http://genome.tugraz.at/genesisclient/genesisclient_description.shtml). On the basis of the more general analysis (Figure 3B), we found suppressors associated with gamma-Tubulin related molecular functions, mitosis and transcription. Enhancers seemed associated with various enzymatic activities and RNA localization, whereas the group of lethal candidates showed diverse immune-responsive functions (regulation of stress-activated protein kinase, RNase complex etc.). The detailed term-by-term analysis of combinations of candidate groups revealed that phenotypic suppressors (categories 1+2) confirmed these findings. On the contrary, enhancers showed relatively weak associations, with the exception of particularly strong enhancers (category 6), which were enriched for RNA localization-related GO terms. The strongest degree of enrichment, however, could be seen for the class of lethal genes (category 7) that showed significant values for many different GO terms, ranging from RNA metabolism and localization to not further specified nuclear functions. The ontology-weighted approach allowed drilling deeper into the GO hierarchy and identifying further functional groups that seem relevant in polyQ-mediated toxicity. Here, enhancers were associated e.g. with axonal growth cone development and splicing-related activities, whereas suppressors showed additional involvement in SH2-domain binding and therefore possibly signal transduction. Again, a very strong degree of GO enrichment was found for the group of lethal genes, with nonsense-mediated decay being one of the strongest terms. Overall, these provide several interesting entry points for further investigations into polyQ-mediated toxicity.

## Discussion

**Figure 4:**
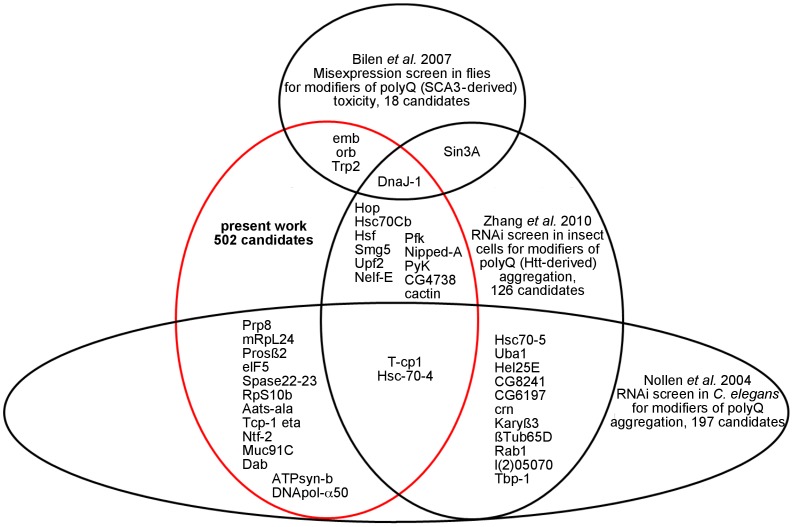
Overlap between screens for genetic modifiers of polyQ-induced toxicity or aggregation. The Venn-like diagram displays only candidate genes shared by the different screens. Mode of modification (enhancement/suppression) is not addressed, due to the different readouts (aggregation/toxicity), model systems (*Drosophila*, insect cells, *C. elegans*) and elongated polyQ-containing proteins used in the diverse screening approaches.

To our knowledge, the present screen for modifiers of polyQ toxicity comprises the largest number of genes analyzed in such assays. Usage of the VDRC RNAi library allows large-scale, almost genome-wide screening. However, RNAi-mediated gene silencing approaches might cause off-target effects. Although the VDRC library was designed to limit off-target effects, we are aware that some of our candidates might result from off-target effects. Additionally, RNAi lines used in this screen were generated by random integrations of UAS-RNAi constructs into the fly genome. Consequently, we cannot exclude the possibility that the site of transgene insertion rather than the RNAi effect itself caused the observed modification on the polyQ-induced REP. In our screen, the plethora of individual RNAi lines and the high number of candidates prevented us to test for potential off-target and/or genetic background effects. Apart of these drawbacks, using RNAi libraries has certain advantages to screen for modifiers of polyQ-induced induced toxicity. For example, previous screens on modifiers of polyQ-induced REPs utilized P-element gene disruption or EP-element-driven overexpression/silencing of genes [18], [19], [20]. Although these screens provided valuable insights in the mechanisms of polyQ-induced toxicity, a drawback of P/EP-element-based screens is the limited amount of available elements and the unknown/low number of targeted genes. The expected low number of assayed genes might explain the small overlap of candidates identified by Bilen and Bonini [18] with our screen (Figure 4). In addition, we compared our data with selected RNAi screens for modifiers of polyQ aggregation performed in cultured insect cells [34] and in *C. elegans* [35]. Although the primary readout has been aggregation rather than toxicity, several common candidates were identified in comparison with our screen. To our surprise, the overlap of the two aggregation screens [34], [35] was as high as with our screen (Figure 4). In a next step, we grouped overlapping candidate genes according to the reported function of their gene products. Almost all common candidates could be assigned to the following three categories: 1. Protein turnover/quality control (*Trp2*, *DnaJ-1*, *Hop*, *Hsc70Cb*, *Hsc70-*4, *Prosß2*, etc); 2. Nuclear import/export (*emb*, *Ntf-2* and *CG5738*) and 3. mRNA transport/editing/translation (*orb*, *Nelf-E*, *Prp8*, etc). These results suggest that impairment of these processes might contribute to disease. This is in line with previous reports showing a strong involvement of the UPS in polyQ toxicity [14], [36], [37], [38], [39], [40]. In addition, network analysis of our candidates implies an enrichment of proteasomal components highlighting the importance of the proteasome in polyQ disease (Figure 3). Moreover, translocation of polyQ peptides into the nucleus is believed to be an important step in disease [23], [28], [29], [41], [42], [43], [44]. Finally, mRNA transport/editing/translation is crucial for cell fitness and tightly regulated. This regulation often takes place in response to or as compensation of cellular stress [45], [46]. The fact that only few of these candidates also had an impact on Tau-induced toxicity, suggests that the regulation of these pathways is rather specific for polyQ-induced toxicity. Heat shock proteins/chaperones like Hsc70-4, Hsc70-1 and Hop are considered to provide protective effects on toxicity exerted by aggregation-prone proteins. Indeed, overexpression of human HSP70 suppresses polyQ toxicity [47], [48]. The tight regulation of heat shock protein (HSPs)/chaperone expression by auto-regulatory mechanisms might explain why silencing of some HSPs suppressed polyQ-induced toxicity. For example, the initiation of HSP transcription by heat stress transcription factor 1 (HSF1) is prevented by direct binding of HSP90 to HSF1 [49].

In agreement with previous reports, our analysis on polyQ aggregation of selected candidates revealed a dissociation of toxicity and aggregation [11], [50], [51]. We found that only a minor portion of analyzed suppressors had a significant effect on polyQ aggregation. More precisely, suppressors caused either a reduction or an increase of aggregated polyQ species compared to control, as visualized by filter retardation assay (Figure 2, Figure S1). We are aware that the filter retardation assay might not perfectly reflect actual aggregate load. According to the pore size of the membrane (0.2 µm), we might not be able to detect aggregates with a diameter smaller than the pore. In addition, we might pellet extremely high molecular weight aggregates by centrifugation steps in sample preparation and thus deplete these aggregate species from our analysis. In case of the analyzed enhancers, there was no clear trend towards increased aggregation (Figure 2B, Figure S1). In contrast, almost all analyzed enhancers displayed a slightly reduced aggregate load. However, the high degree of retina cell loss observed for enhancers might bias the actual aggregate load due to a reduction in the absolute number of polyQ-expressing cells present at the time of analysis. In summary, our findings nevertheless imply absence of correlation between toxicity and aggregation. This was at least partially unexpected as previous analyses implicated such a correlation and convincingly proved this assumption with a wide range of experimental approaches [52], [53]. A smaller sample number in previous reports might account for the discrepancy compared to our analysis.

The computational analysis of our candidate gene set highlights the broad range of molecular functions that might affect polyQ-mediated toxicity. The network-based approach utilizes subtle phenotypic changes of some candidates to tie links between strong candidate genes. While not all subtle candidates may be ‘true’, a good proportion actually does make sense in the light of the network- and Gene Ontology analysis. A future challenge will be the identification and assessment of the most important functional categories that might moderate polyQ-induced toxicity.

## Methods

**Flies** were raised and maintained on standard cornmeal-agar-yeast food. If not stated otherwise, all crosses were performed at 25°C. The “human ortholog RNAi library” (status October 2007) was obtained from the Vienna *Drosophila* RNAi Center (VDRC). Selection of human orthologs was done by the VDRC using common databases. Filter criteria were not provided. RNAi lines for confirmation were provided by the Bloomington *Drosophila* Stock Center (BDSC, USA) or the National Institute of Genetics (NIG-fly, Japan). Non-RNAi lines: w[*]; P{w[+mC] = UAS-Hsap\MJD.tr-Q78}c211.2 (BDSC 8150; allows expression of HA-tagged C-terminal fragment of Ataxin-3 with a 78 repeat polyQ tract; referred to in text as *polyQ*); w[*]; P{w[+mC] = longGMR-GAL4} (BDSC 8605; referred to as *GMR-GAL4* in text). Additional fly strains used: w[*]; P{Act5C-GAL4}/CyO driver (*Act-GAL4* in text, provided by the Herbert Jäckle laboratory), *P{w[+mW.hs] = GawB}elav[C155]* (BDSC 458, *elav-GAL4* in text) and w[*];; P{w[+mC] = UAS-hTau[R406W]} (kindly provided by Mel Feany).

**Screening** was performed using flies in which the *GMR-GAL4* driver was recombined with the *polyQ* transgene (w[*]; P{w[+mC] = longGMR-GAL4}, P{w[+mC] = UAS-Hsap\MJD.tr-Q78}c211.2/CyO; *GMR>polyQ* in text). *GMR>polyQ* virgins were crossed to males carrying UAS-RNAi constructs. F1 females (*GMR*>*polyQ* in combination with respective UAS-RNAi expression) were selected for REP evaluation 1–5 days post eclosion. Effects on the polyQ-induced REP were categorized in following groups:

(1) wildtype-like suppression, (2) robust suppression, (3) subtle suppression, (4) no change, (5) subtle enhancement, (6) robust enhancement, and (7) lethal.

Only strong modifiers (categories 1, 2, 6, 7) were verified thrice and then considered as candidates. Subtle modifiers were only included in computational analyses.

**Rescue** of lethality following pan-neural polyQ expression was assayed at 29°C. In a first step, *elav-GAL4* virgins with balanced 2^nd^ (*Sco/CyO*) or 3^rd^ (*CxD/TM3*) chromosomes were crossed to flies harboring respective 2^nd^ or 3^rd^ chromosomal UAS-RNAi transgenes. In the F1 generation, males carrying *elav-GAL4* in combination with balanced UAS-RNAi transgenes (*elav-GAL4/Y; UAS-RNAi/CyO or elav-GAL4/Y;; UAS-RNAi/TM3*) were selected and crossed to homozygous *polyQ* virgins. Presence of female offspring was monitored in the F2 generation.

**Filter retardation assays** for evaluation of polyQ aggregate load were mainly conducted as described [31], [53]. Briefly, fly heads were lysed in RIPA buffer (50 mM Tris, pH 8.0, 0.15 M NaCl, 0.1% (v/v) SDS, 1% NP-40, 0.5% Sodium deoxycholate, Protease inhibitor (Roche)). 15 µg protein from fly head homogenates (DC Protein Assay Kit, BIO-RAD) were subjected with 1× dot blot buffer (20% (v/v) Glycerol, 0.2 M DTT, TRIS-HCl, pH 6.8) and boiled (5 min). Using a dot blot filtration unit, lysates were filtered through a nitrocellulose membrane (Whatman, pore size 0.2 µm) equilibrated with 0.1% SDS in TBS (25 mM Tris, 140 mM NaCl, pH 7.5) and afterwards washed in TBS+0.05% Tween-20. The membrane was probed with mouse anti-HA antibody (Covance, 1∶1,000) and secondary HRP-coupled antibody (GE Healthcare, 1∶10,000). ECL solution was used for visualization. Independent homogenates (if available) were used for repetitions. In case of none or only one independent repetition n≤2 is indicated. In all other cases, number of independent repetitions is n≥3. In addition, regular Western blots of head lysates were probed with Syntaxin antibody (DSHB 8C3 1∶2,500) to control for equal protein loading. Statistics: Variation within the data set was tested independently for suppressors and enhancers with ANOVA. If variation was significant, Bonferroni post-hoc test was applied (GraphPad Prism 5).

**Computational analyses** were performed primarily with custom-written Perl scripts. The network graph on the basis of the meta-interaction network [32] was generated using Cytoscape v2.8 [54]. Gene Ontology over-representation statistics were calculated using the command line version of Ontologizer v2.0 [55], using the set of tested RNAi lines as background population. The resulting matrix of candidate gene groups and Gene Ontology terms was clustered and displayed using Genesis v1.76 [56].

## Supporting Information

Figure S1 **Filter retardation and Western blot analysis of selected head lysates.** Filter retardation assay (FRA) was used to visualize polyQ aggregates. Western blot (WB) analysis of the head lysates to monitor abundance of Syntaxin was used for normalization purposes. Transformant IDs of selected suppressors and enhancers of polyQ-induced REPs are indicated.
(TIF)

Figure S2 **Gene Ontology analysis of candidate gene groups.** Shown are −log_10_(p-value) scores for GO term enrichment for each non-redundant combination of candidate gene groups (horizontal axis) and GO term (vertical). The analysis incorporated all possible combinations of subtle, strong and lethal candidate groups. The range of phenotypes was categorized: 1 full, 2 robust and 3 subtle suppression of REP, 5 subtle and 6 robust enhancement of REP, 7 indicating lethality. The upper matrix is based on simple term by term comparison for GO term enrichment with a Benjamini/Hochberg-corrected p-value<0.15. While the first approach yielded vastly redundant terms of primarily nuclear processes, the latter approach (Topology Weighted-annotation considering the tree hierarchy of the ontology, lower matrix) uncovered potential molecular functions as distinct as splicing and transmembrane receptor signaling.
(TIF)

Table S1 **Identified obvious modifiers of the SCA3tr-Q78-induced REP.** Table lists transformant ID (from VDRC), gene ID and gene name (if applicable) of all candidates identified along with the observed effects on the SCA3-induced phenotype: wildtype-like suppression (S*), robust suppression (S), robust enhancement (E), or lethal interaction (lethal). PolyQ modifiers with similar effects on Tau[R406W]-induced toxicity are highlighted in grey. Essential genes with amorphic mutations known to cause lethality are indicated (§). Reduced vitality or lethality following ubiquitous shRNA (*actin5C-GAL4*) against these genes is indicated in red. Lines not available for re-screening and/or photographs are marked as not analyzed (n.a.).
(DOC)

Table S2 **Rescue of lethality induced by pan-neural polyQ expression.** Table lists transformant ID (from VDRC), gene ID and gene name (if applicable) of all RNAi lines (genes silenced) which were tested for rescue effects on *elav>polyQ*-induced lethality. In the F1 generation (*elav>polyQ* in combination with respective RNAi line), effects of gene silencing were categorized as rescue (R) if vital offspring was observed, or lethal (L) if no vital offspring was present. Control (*white* RNAi) is marked in grey. Lines not available for rescue experiments are marked as not analyzed (n.a.).
(DOC)

Dataset S1 **Raw data archive in ZIP format.** Supplementary File F1.cys for visualization in Cytoscape contains a network graph with RNAi screen candidates mapped onto the 20 k network of Costello et al. 2009. Primary candidates are represented in red, subtle candidates in black. The two Term2TermGOTerms as well as the two TopologyWeightedGOTerms files contain GO enrichment statistics and clustering results, and can be directly loaded into Genesis for visualization.
(ZIP)
