# Supplementary figures and images for: Large-Scale Screen for Modifiers of Ataxin-3-Derived Polyglutamine-Induced Toxicity in Drosophila
Source: PLoS One. 2012 Nov 5;7(11):e47452. doi: 10.1371/journal.pone.0047452 (PMC3489908; doi:10.1371/journal.pone.0047452)

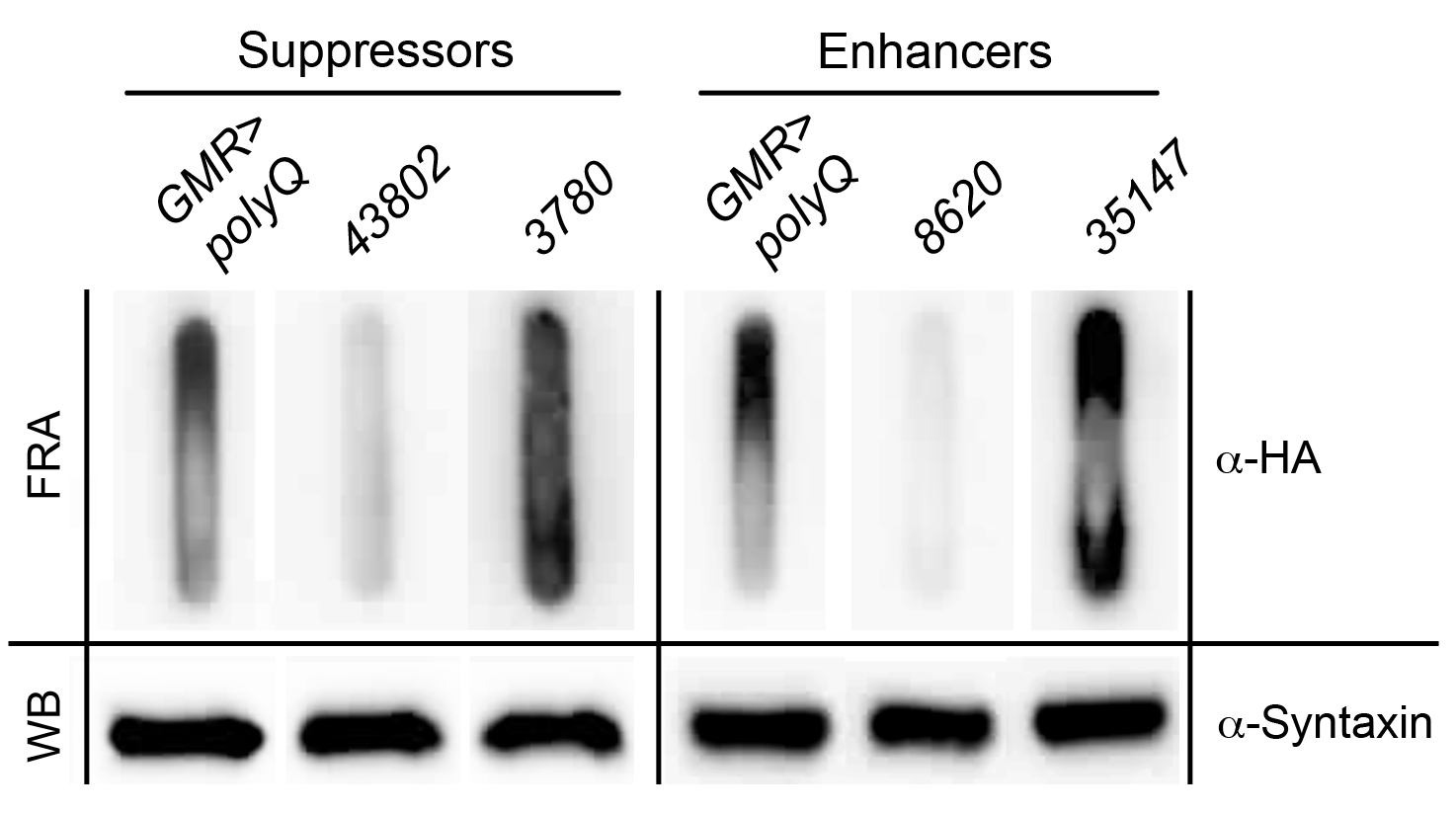

Supplement: Figure S1 — Filter retardation and Western blot analysis of selected head lysates. Filter retardation assay (FRA) was used to visualize polyQ aggregates. Western blot (WB) analysis of the head lysates to monitor abundance of Syntaxin was used for normalization purposes. Transformant IDs of selected suppressors and enhancers of polyQ-induced REPs are indicated. (TIF) [file pone.0047452.s001.tif]

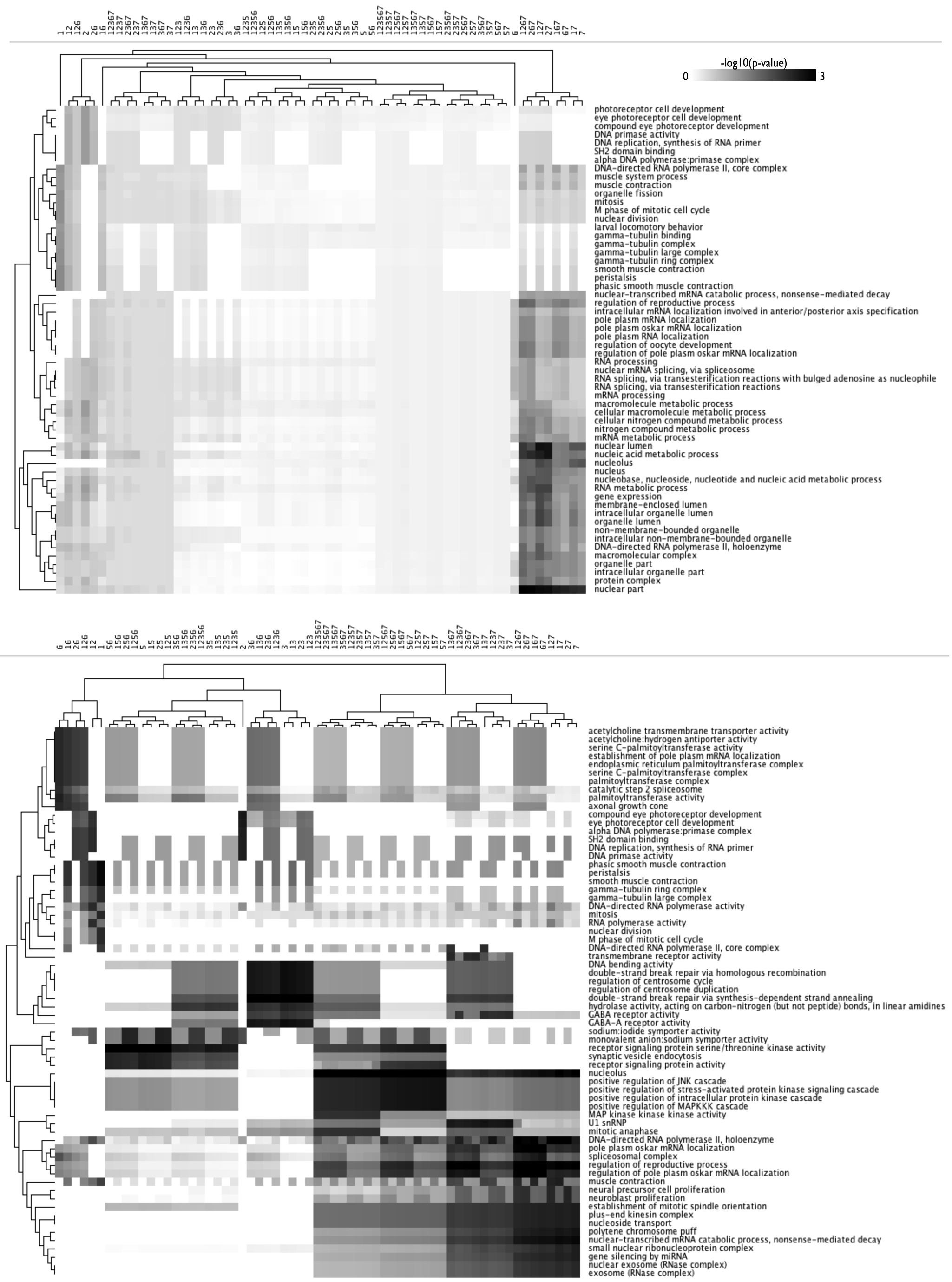

Supplement: Figure S2 — Gene Ontology analysis of candidate gene groups. Shown are −log10(p-value) scores for GO term enrichment for each non-redundant combination of candidate gene groups (horizontal axis) and GO term (vertical). The analysis incorporated all possible combinations of subtle, strong and lethal candidate groups. The range of phenotypes was categorized: 1 full, 2 robust and 3 subtle suppression of REP, 5 subtle and 6 robust enhancement of REP, 7 indicating lethality. The upper matrix is based on simple term by term comparison for GO term enrichment with a Benjamini/Hochberg-corrected p-value<0.15. While the first approach yielded vastly redundant terms of primarily nuclear processes, the latter approach (Topology Weighted-annotation considering the tree hierarchy of the ontology, lower matrix) uncovered potential molecular functions as distinct as splicing and transmembrane receptor signaling. (TIF) [file pone.0047452.s002.tif]
