# Supplementary material for: Large-Scale Screen for Modifiers of Ataxin-3-Derived Polyglutamine-Induced Toxicity in Drosophila
Source: PLoS One. 2012 Nov 5;7(11):e47452. doi: 10.1371/journal.pone.0047452 (PMC3489908; doi:10.1371/journal.pone.0047452)
Supplement: Table S1 — Identified obvious modifiers of the SCA3tr-Q78-induced REP. Table lists transformant ID (from VDRC), gene ID and gene name (if applicable) of all candidates identified along with the observed effects on the SCA3-induced phenotype: wildtype-like suppression (S*), robust suppression (S), robust enhancement (E), or lethal interaction (lethal). PolyQ modifiers with similar effects on Tau[R406W]-induced toxicity are highlighted in grey. Essential genes with amorphic mutations known to cause lethality are indicated (§). Reduced vitality or lethality following ubiquitous shRNA (actin5C-GAL4) against these genes is indicated in red. Lines not available for re-screening and/or photographs are marked as not analyzed (n.a.). (DOC) [file pone.0047452.s003.doc]

**Supplementary Table S1: Identified obvious modifi**ers of the SCA3tr-Q78-induced REP.

| **Transformant ID** | **CG number** | | | **Gene name** | **REP**  **modification** | | **Phenotype** |
| --- | --- | --- | --- | --- | --- | --- | --- |
| 8780 | CG17048 | | | CG17048 | S* | | 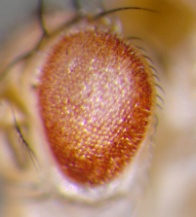 |
| 7903 | CG9501 | | | ppk14 | S* | | 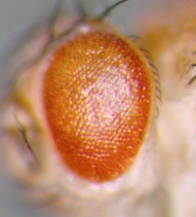 |
| 23121 | CG7123 | | | LanB1 | S* | | n.a. |
| 44362 | CG9131 | | | slmo | S* | | n.a. |
| 37221 | CG9153 | | | CG9153 | S* | | 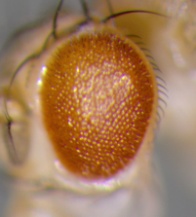 |
| 15789 | CG8696 | | | LvpH | S* | | 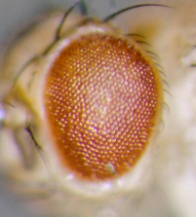 |
| 26465 | CG4264 | | | Hsc70-4 | S* | | 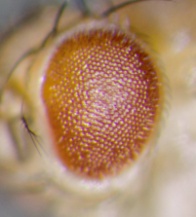 |
| 50222 | CG4264 | | | Hsc70-4 | S* | | 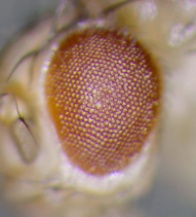 |
| 11219 | CG3284 | | | RpII15 | S* | | 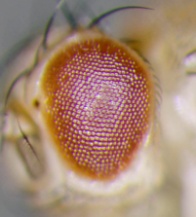 |
| 3780 | CG5799 | | | dve-s | S* | | 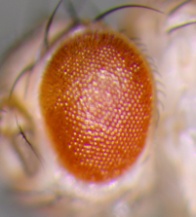 |
| 24030 | CG9448 | | | trbd | S | | 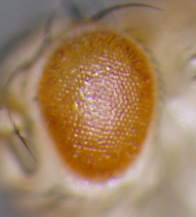 |
| 40006 | CG15618 | | | CG15618 | S | | 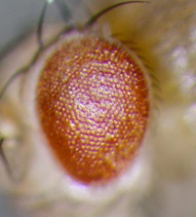 |
| 41530 | CG14514 | | | Brd8 | S | | 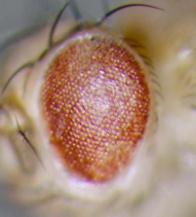 |
| 23637 | CG8863 | | | Droj2 | S | | 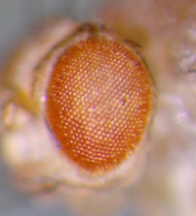 |
| 43870 | CG7108 | | | DNApol-alpha50 | S | | 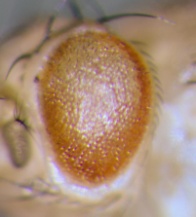 |
| 45596 | CG8937 | | | Hsc70-1 | S | | 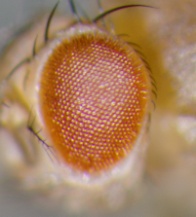 |
| 41696 | CG2720 | | | Hop | S | | 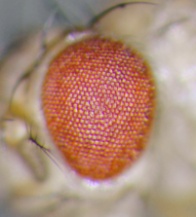 |
| 40044 | CG16890 | | | CG16890 | S | | 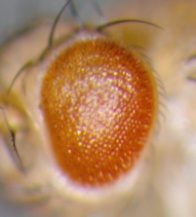 |
| 8070 | CG13969 | | | bwa | S | | 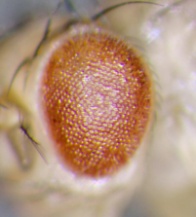 |
| 6282 | CG6755 | | | EloA | S | | 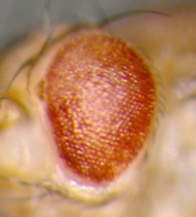 |
| 31257 | CG10545 | | | Gbeta13F | S | | 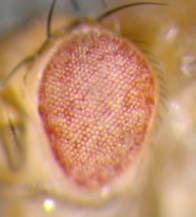 |
| 19066 | CG1658 | | | Doa | S | | n.a. |
| 33262 | CG5687 | | | CG5687 | S | | 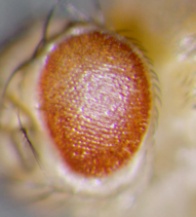 |
| 13005 | CG9695 | | | Dab | S | | 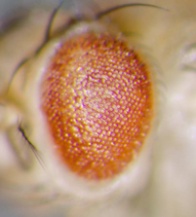 |
| 16182 | CG1107 | | | aux | S | | 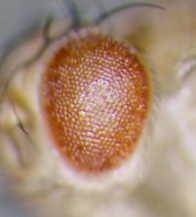 |
| 37930 | CG14619 | | | CG14619 | S | | 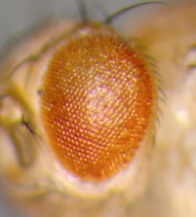 |
| 46473 | CG17919 | | | CG17919 | S | | 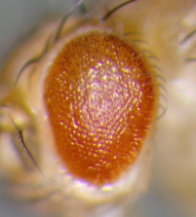 |
| 43606 | CG6758 | | | CG6758 | S | | 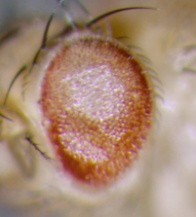 |
| 43802 | CG6363 | | | MRG15 | S | | 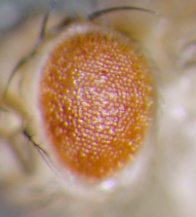 |
| 8408 | CG3389 | | | Cad88C | S | | 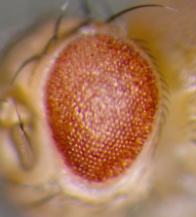 |
| 34713 | CG3808 | | | CG3808 | S | | 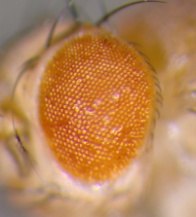 |
| 25030 | CG31110 | | | 5PtaseI | S | | 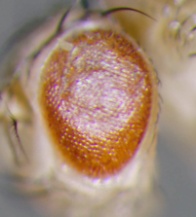 |
| 26475 | CG4266 | | | CG4266 | S | | 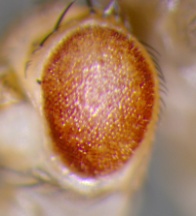 |
| 23843 | CG16807 | | | roq | S | | 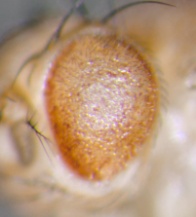 |
| 16040 | CG10377 | | | Hrb27C | S | | 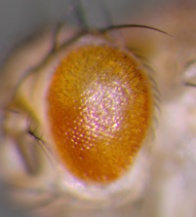 |
| 17196 | CG13467 | | | CG42247 | S | | n.a. |
| 22590 | CG7855 | | | timeout | E | | 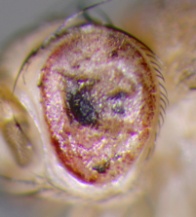 |
| 24070 | CG9601 | | | CG9601 | E | | 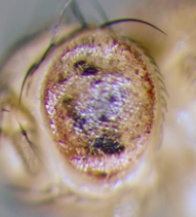 |
| 30186 | CG15534 | | | CG15534 | E | | 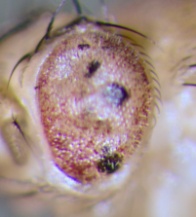 |
| 1326 | CG10001 | | | AR-2 | E | | 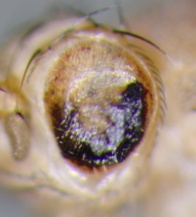 |
| 47569 | CG12935 | | | CG12935 | E | | 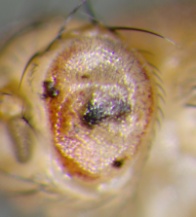 |
| 21293 | CG31048 | | | CG31048 | E | | 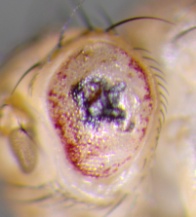 |
| 10942 | CG1119 | | | Gnf1 | E | | n.a. |
| 48062 | CG1695 | | | CG1695 | E | | 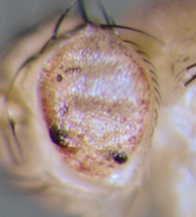 |
| 28386 | CG4881 | | | salr | E | | n.a. |
| 36025 | CG8781 | | | tsu | E | | n.a. |
| 22454 | CG6873 | | | CG6873 | E | | 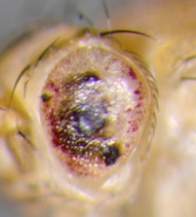 |
| 41960 | CG3799 | | | Exn | E | | n.a. |
| 31777 | CG13298 | | | CG13298 | E | | n.a. |
| 43612 | CG14966 | | | CG14966 | E | | 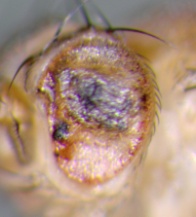 |
| 24885 | CG14622 | | | DAAM | E | | 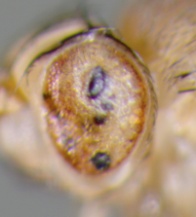 |
| 40478 | CG3869 | | | Marf | E | | 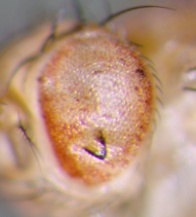 |
| 29711 | CG6115 | | | CG6115 | E | | n.a. |
| 5684 | CG5229 | | | chm | E | | 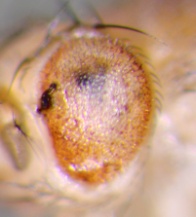 |
| 42798 | CG6627 | | | Dnz1 | E | | 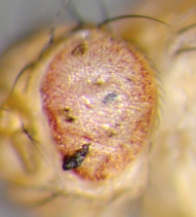 |
| 49792 | CG3678 | | | CG3678 | E | | n.a. |
| 30717 | CG10872 | | | CG33128 | E | | 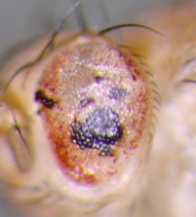 |
| 23659 | CG8954 | | | Smg5 | E | | 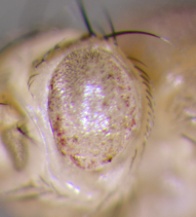 |
| 33581 | CG2887 | | | CG2887 | E | | n.a. |
| **48692** | **CG5748 §** | | | **Hsf** | **E** | | 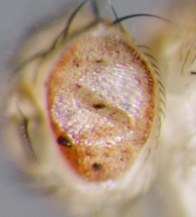 |
| 35147 | CG6930 | | | l(3)neo38 | E | | 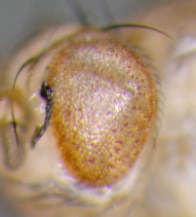 |
| 44114 | CG11722 | | | CG11722 | E | | 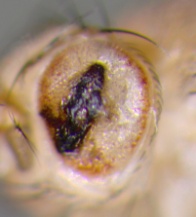 |
| 19450 | CG15399 | | | CG15399 | E | | 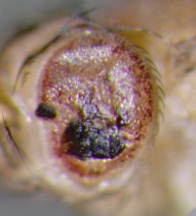 |
| 24725 | CG3225 | | | CG3225 | E | | n.a. |
| 20536 | CG17753 | | | CCS | E | | 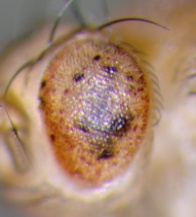 |
| 10020 | CG4016 | | | Spt-I | E | | n.a. |
| 32370 | CG31641 | | | stai | E | | n.a. |
| 36153 | CG18679 | | | CG34372 | E | | 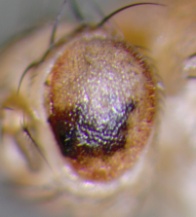 |
| 20183 | CG12345 | | | Cha | E | | n.a. |
| 36572 | CG7066 | | | Sbp2 | E | | 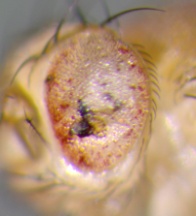 |
| 33837 | CG10524 | | | Pkcdelta | E | | 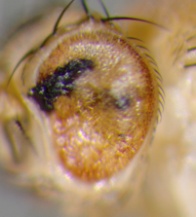 |
| 8620 | CG4288 | | | CG4288 | E | | 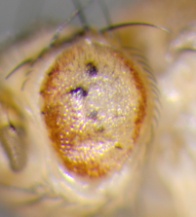 |
| 28019 | CG7436 §^n.a^. | | | Nmt | lethal | |  |
| 22574 | CG7843 | | | Ars2 | lethal | |  |
| 51209 | CG10281 | | | TfIIFalpha | lethal | |  |
| 14874 | CG2145 | | | CG2145 | lethal | |  |
| 14890 | CG15739 | | | CG15739 | lethal | |  |
| 22561 | CG7275 | | | CG7275 | lethal | |  |
| 30179 | CG6921 | | | bond | lethal | |  |
| 30214 | CG16785 | | | fz3 | lethal | |  |
| 21393 | CG31687 | | | CG31687 | lethal | |  |
| 1385 | CG9753 | | | AdoR | lethal | |  |
| 9865 | CG7709 | | | Muc91C | lethal | |  |
| **41130** | **CG7807 §** | | | **AP-2** | **lethal** | |  |
| **46150** | **CG7085 §** | | | **l(2)s5379** | **lethal** | |  |
| 21985 | CG31318 | | | Rpb4 | lethal | |  |
| 38471 | CG1129 | | | CG1129 | lethal | |  |
| **45635** | **CG6944 §** | | | **Lam** | **lethal** | |  |
| 46072 | CG6589 | | | spag4 | lethal | |  |
| 26959 | CG8431 | | | Aats-cys | lethal | |  |
| 44942 | CG31321 | | | CG31321 | lethal | |  |
| 40907 | CG5404 | | | CG5404 | lethal | |  |
| 39402 | CG5310 | | | nmdyn-D6 | lethal | |  |
| 14210 | CG8189 | | | ATPsyn-b | lethal | |  |
| 14194 | CG17081 | | | Cep135 | lethal | |  |
| 39224 | CG18812 | | | CG18812 | lethal | |  |
| 39256 | CG9742 | | | SmG | lethal | |  |
| 20334 | CG16938 | | | Tif-IA | lethal | |  |
| 18107 | CG7279 | | | Lip1 | lethal | |  |
| 32680 | CG1640 | | | CG1640 | lethal | |  |
| 44991 | CG11136 | | | Lrt | lethal | |  |
| 16506 | CG5599 | | | CG5599 | lethal | |  |
| 28745 | CG12524 | | | CG34356 | lethal | |  |
| 34160 | CG6340 | | | CG6340 | lethal | |  |
| 50706 | CG4241 | | | att-ORFA | lethal | |  |
| 13613 | CG2917 | | | Orc4 | lethal | |  |
| 35065 | CG6066 | | | CG6066 | lethal | |  |
| 36050 | CG8849 | | | mRpL24 | lethal | |  |
| 21792 | CG4132 | | | pkaap | lethal | |  |
| 40076 | CG17419 | | | CG41099 | lethal | |  |
| 21793 | CG4152 §^n.a^. | | | l(2)35Df | lethal | |  |
| 11852 | CG9245 | | | Pis | lethal | |  |
| 15602 | CG3759 | | | CG3759 | lethal | |  |
| 47537 | CG11010 | | | Ent3 | lethal | |  |
| 49822 | CG9958 | | | snapin | lethal | |  |
| 6143 | CG4928 | | | CG4928 | lethal | |  |
| 40972 | CG17681 | | | CG17681 | lethal | |  |
| 43998 | CG6335 | | | Aats-his | lethal | |  |
| 44104 | CG10126 | | | CG10126 | lethal | |  |
| 39848 | CG14905 | | | CG14905 | lethal | |  |
| 21308 | CG31291 | | | CG31291 | lethal | |  |
| **33735** | **CG31000 §** | | | **heph** | **lethal** | |  |
| 33787 | CG31211 | | | CG31211 | lethal | |  |
| 25195 | CG15143 | | | CG15143 | lethal | |  |
| 14869 | CG2124 | | | CG2124 | lethal | |  |
| 29332 | CG9927 | | | Art6 | lethal | |  |
| 14861 | CG7598 | | | CG7598 | lethal | |  |
| 34316 | CG5121 | | | MED28 | lethal | |  |
| 38269 | CG3776 | | | CG3776 | lethal | |  |
| **33186** | **CG4482 §** | | | **mol** | **lethal** | |  |
| **21139** | **CG3035 §** | | | **cm** | **lethal** | |  |
| 38963 | CG7935 | | | msk | lethal | |  |
| 49153 | CG13779 | | | CG13779 | lethal | |  |
| 24177 | CG9998 §^n.a^. | | | U2af50 | lethal | |  |
| 38491 | CG11360 | | | CG11360 | lethal | |  |
| 39450 | CG31704 | | | CG31704 | lethal | |  |
| 34479 | CG32253 | | | CG11583 | lethal | |  |
| 21999 | CG5085 | | | Sirt2 | lethal | |  |
| 22068 | CG5335 | | | CG5335 | lethal | |  |
| 31789 | CG13779 | | | CG13779 | lethal | |  |
| 33561 | CG2708 | | | Tom34 | lethal | |  |
| 49879 | CG7014 | | | RpS5b | lethal | |  |
| 3245 | CG10913 | | | Spn6 | lethal | |  |
| 26432 | CG4202 | | | Sas10 | lethal | |  |
| 6723 | CG10693 | | | slo | lethal | |  |
| **30623** | **CG5553 §** | | | **DNApol-alpha60** | **lethal** | |  |
| 49525 | CG33505 | | | U3-55K | lethal | |  |
| 51979 | CG10564 | | | Ac78C | lethal | |  |
| **7802** | **CG8933 §** | | | **exd** | **lethal** | |  |
| 52165 | CG13849 | | | Nop56 | lethal | |  |
| **7800** | **CG4035 §** | | | **eIF-4E** | **lethal** | |  |
| 51363 | CG16884 | | | CG16884 | lethal | |  |
| 4634 | CG30048 | | | CG30048 | lethal | |  |
| 52094 | CG10582 | | | Sin | lethal | |  |
| 46977 | CG1989 | | | Yippee | lethal | |  |
| 44976 | CG7769 §^n.a^. | | | pic | lethal | |  |
| 27610 | CG4969 | | | Wnt6 | lethal | |  |
| **42915** | **CG7176 §** | | | **Idh** | **lethal** | |  |
| **21374** | **CG3158 §** | | | **spn-E** | **lethal** | |  |
| 42716 | CG5911 | | | ETHR | lethal | |  |
| 2487 | CG17075 | | | CG17075 | lethal | |  |
| 8361 | CG11278 §^n.a^. | | | Syx13 | lethal | |  |
| 7787 | CG9696 | | | dom | lethal | |  |
| 34145 | CG5869 | | | CG5869 | lethal | |  |
| 38319 | CG10033 §^n.a^. | | | for | lethal | |  |
| 41406 | CG1882 | | | CG1882 | lethal | |  |
| 44562 | CG11739 | | | CG11739 | lethal | |  |
| **45402** | **CG12298** | | | **sub** | **lethal** | |  |
| 1335 | CG11958 | | | Cnx99A | lethal | |  |
| 8907 | CG1139 | | | CG1139 | lethal | |  |
| **24749** | **CG3329 §** | | | **Prosbeta2** | **lethal** | |  |
| 49245 | CG2241 | | | Rpt6R | lethal | |  |
| 7308 | CG3305 | | | CG3305 | lethal | |  |
| 52486 | CG2905 | | | Nipped-A | lethal | |  |
| **9039** | **CG6827 §** | | | **Nrx-IV** | **lethal** | |  |
| 2857 | CG7431 | | | CG7431 | lethal | |  |
| 18440 | CG2918 | | | CG2918 | lethal | |  |
| **31522** | **CG11546 §** | | | **kermit** | **lethal** | |  |
| 44325 | CG5651 | | | pix | lethal | |  |
| 44589 | CG12275 | | | RpS10a | lethal | |  |
| 47116 | CG5969 | | | CG5969 | lethal | |  |
| 17171 | CG13391 | | | Aats-ala | lethal | |  |
| 12482 | CG2478 | | | bru | lethal | |  |
| **46499** | **CG1030 §** | | | **Scr** | **lethal** | |  |
| 38154 | CG3589 | | | CG3589 | lethal | |  |
| 39091 | CG31289 | | | Dph5 | lethal | |  |
| 22496 | CG6509 | | | CG6509 | lethal | |  |
| 37250 | CG5751 | | | TrpA1 | lethal | |  |
| 28341 | CG1903 §^n.a^. | | | sno | lethal | |  |
| 27152 | CG10315 | | | eIF2B-delta | lethal | |  |
| 40477 | CG3843 | | | RpL10Aa | lethal | |  |
| 13054 | CG7162 | | | MED1 | lethal | |  |
| 52392 | CG4960 | | | CG4960 | lethal | |  |
| 47126 | CG3849 | | | Lasp | lethal | |  |
| 33135 | CG4521 | | | mthl1 | lethal | |  |
| 33256 | CG3499 | | | CG3499 | lethal | |  |
| 25246 | CG17293 | | | CG17293 | lethal | |  |
| 15877 | CG12031 | | | MED14 | lethal | |  |
| **10639** | **CG6146 §** | | | **Top1** | **lethal** | |  |
| 25547 | CG7757 | | | CG7757 | lethal | |  |
| 17302 | CG12727 | | | CG32635 | lethal | |  |
| 25535 | CG7742 | | | CG7742 | lethal | |  |
| **42010** | **CG4843 §** | | | **Tm2** | **lethal** | |  |
| 49800 | CG6835 | | | GS | lethal | |  |
| 23689 | CG9344 | | | CG9344 | lethal | |  |
| 36252 | CG12283 | | | kek1 | lethal | |  |
| 31726 | CG12325 | | | CG12325 | lethal | |  |
| 13503 | CG8222 | | | Pvr | lethal | |  |
| 30000 | CG4357 | | | Ncc69 | lethal | |  |
| 41980 | CG4180 §^n.a^. | | | l(2)35Bg | lethal | |  |
| 36175 | CG9961 | | | CG9961 | lethal | |  |
| 30462 | CG6534 | | | slou | lethal | |  |
| 36121 | CG9619 | | | CG9619 | lethal | |  |
| 30448 | CG5179 | | | Cdk9 | lethal | |  |
| 30431 | CG7772 | | | CG7772 | lethal | |  |
| **34618** | **CG3431 §** | | | **Uch-L3** | **lethal** | |  |
| 41977 | CG4165 | | | CG4165 | lethal | |  |
| 41965 | CG30000 | | | CG30000 | lethal | |  |
| 35200 | CG7292 | | | Rrp6 | lethal | |  |
| 34845 | CG4438 | | | CG4438 | lethal | |  |
| 12375 | CG12318 | | | CG33121 | lethal | |  |
| 13828 | CG7636 | | | mRpL2 | lethal | |  |
| 14972 | CG16812 | | | CG16812 | lethal | |  |
| 40789 | CG10549 | | | Nipped-A | lethal | |  |
| 4426 | CG14511 | | | CG14511 | lethal | |  |
| 11765 | CG8727 | | | cyc | lethal | |  |
| 15547 | CG9271 | | | Vm34Ca | lethal | |  |
| 49979 | CG17664 | | | CG17664 | lethal | |  |
| 13643 | CG10898 | | | CG10898 | lethal | |  |
| 44201 | CG3876 | | | CG3876 | lethal | |  |
| 3579 | CG7899 | | | Acph-1 | lethal | |  |
| 34377 | CG7222 | | | CG7222 | lethal | |  |
| 41599 | CG1578 | | | CG1578 | lethal | |  |
| **17490** | **CG1433 §** | | | **Atu** | **lethal** | |  |
| 45116 | CG11454 | | | CG11454 | lethal | |  |
| 42018 | CG5018 | | | CG5018 | lethal | |  |
| 50797 | CG17949 | | | His2B:CG17949 | lethal | |  |
| 27498 | CG5735 | | | orb2 | lethal | |  |
| 15261 | CG13926 | | | CG13926 | lethal | |  |
| 18567 | CG8877 | | | Prp8 | lethal | |  |
| 25787 | CG31657 | | | PNUTS | lethal | |  |
| 31456 | CG11201 | | | TTLL3B | lethal | |  |
| 49547 | CG31201 | | | GluRIIE | lethal | |  |
| 50510 | CG33931 | | | Rpp20 | lethal | |  |
| 32443 | CG31639 | | | Uch-L3 | lethal | |  |
| 34995 | CG5394 | | | Aats-glupro | lethal | |  |
| 7752 | CG5353 | | | Aats-thr | lethal | |  |
| 29589 | CG3071 | | | CG3071 | lethal | |  |
| 23033 | CG8091 | | | Nc | lethal | |  |
| 13044 | CG1271 | | | CG1271 | lethal | |  |
| 41714 | CG7650 | | | CG7650 | lethal | |  |
| 26001 | CG6852 | | | CG6852 | lethal | |  |
| 11693 | CG6364 | | | CG6364 | lethal | |  |
| **26759** | **CG10726 §** | | | **barr** | **lethal** | |  |
| 5322 | CG5582 | | | cln3 | lethal | |  |
| 26007 | CG7039 | | | CG7039 | lethal | |  |
| 7878 | CG7234 | | | GluRIIB | lethal | |  |
| **10756** | **CG10037 §** | | | **vvl** | **lethal** | |  |
| **49844** | **CG14206 §** | | | **RpS10b** | **lethal** | |  |
| 46284 | CG15772 | | | CG15772 | lethal | |  |
| 17002 | CG10811 | | | eIF4G | lethal | |  |
| 43955 | CG1412 | | | RhoGAP19D | lethal | |  |
| 3347 | CG13387 | | | emb | lethal | |  |
| 44557 | CG6707 | | | CG6707 | lethal | |  |
| 28065 | CG7788 | | | Ice | lethal | |  |
| 28798 | CG9924 | | | rdx | lethal | |  |
| 45530 | CG4751 | | | CG4751 | lethal | |  |
| 4047 | CG12891 | | | CPTI | lethal | |  |
| **44263** | **CG7480 §** | | | **Pgant35A** | **lethal** | |  |
| 44535 | CG4780 | | | membrin | lethal | |  |
| 46445 | CG33193 | | | sav | lethal | |  |
| 44570 | CG9867 | | | CG9867 | lethal | |  |
| 49372 | CG11877 | | | CG11877 | lethal | |  |
| 12209 | CG2901 | | | CG2901 | lethal | |  |
| 38481 | CG11299 | | | CG11299 | lethal | |  |
| 45789 | CG8258 | | | CG8258 | lethal | |  |
| **12746** | **CG5163 §** | | | **TfIIA-S** | **lethal** | |  |
| **12645** | **CG1064 §** | | | **Snr1** | **lethal** | |  |
| 39207 | CG9802 | | | Cap | lethal | |  |
| 22480 | CG6226 | | | FK506-bp1 | lethal | |  |
| 18031 | CG10192 | | | eIF4G2 | lethal | |  |
| 49655 | CG10546 | | | Cralbp | lethal | |  |
| 38399 | CG10716 | | | 4EHP | lethal | |  |
| 23873 | CG13349 | | | CG13349 | lethal | |  |
| **44484** | **CG1616 §** | | | **dpa** | **lethal** | |  |
| 23851 | CG1316 | | | CG1316 | lethal | |  |
| 44449 | CG1718 | | | CG1718 | lethal | |  |
| 40336 | CG5913 | | | CG5913 | lethal | |  |
| **4789** | **CG10975 §** | | | **Ptp69D** | **lethal** | |  |
| 10843 | CG9426 | | | CG9426 | lethal | |  |
| **31619** | **CG11989 §** | | | **Ard1** | **lethal** | |  |
| 21010 | CG5994 | | | Nelf-E | lethal | |  |
| 842 | CG14396 | | | Ret | lethal | |  |
| 31444 | CG11184 | | | Upf3 | lethal | |  |
| **12768** | **CG5499 §** | | | **His2Av** | **lethal** | |  |
| 21258 | CG3058 | | | Dim1 | lethal | |  |
| 23625 | CG8841 | | | CG8841 | lethal | |  |
| 19208 | CG3011 | | | CG3011 | lethal | |  |
| 32719 | CG1676 | | | cactin | lethal | |  |
| 15627 | CG10984 | | | CG10984 | lethal | |  |
| 43790 | CG5405 | | | KrT95D | lethal | |  |
| 43549 | CG11899 | | | CG11899 | lethal | |  |
| 50643 | CG31809 | | | CG31809 | lethal | |  |
| 3326 | CG10657 | | | CG10657 | lethal | |  |
| 34792 | CG4090 | | | Mur89F | lethal | |  |
| **6315** | **CG8384 §** | | | **gro** | **lethal** | |  |
| **20876** | **CG2503 §** | | | **atms** | **lethal** | |  |
| **865** | **CG10776 §** | | | **wit** | **lethal** | |  |
| 35272 | CG7791 | | | CG7791 | lethal | |  |
| 36028 | CG8786 | | | CG8786 | lethal | |  |
| 26615 | CG4735 | | | shu | lethal | |  |
| **12581** | **CG8151 §** | | | **Tfb1** | **lethal** | |  |
| 12149 | CG7623 | | | sll | lethal | |  |
| 49328 | CG11907 | | | Ent1 | lethal | |  |
| **15453** | **CG3644 §** | | | **bic** | **lethal** | |  |
| 50435 | CG32602 | | | Muc12Ea | lethal | |  |
| 30884 | CG10374 | | | Lsd-1 | lethal | |  |
| 32395 | CG16901 | | | sqd | lethal | |  |
| 4180 | CG12929 | | | CG12929 | lethal | |  |
| 16125 | CG10961 | | | Traf6 | lethal | |  |
| 49848 | CG1740 | | | Ntf-2 | lethal | |  |
| 37663 | CG5640 | | | Utx | lethal | |  |
| 7748 | CG7926 | | | Axn | lethal | |  |
| 15185 | CG5186 | | | slim | lethal | |  |
| 22548 | CG7257 | | | Rpt4R | lethal | |  |
| 29253 | CG13628 | | | Rpb10 | lethal | |  |
| 42779 | CG3881 | | | GlcAT-S | lethal | |  |
| 16569 | CG12951 | | | CG12951 | lethal | |  |
| 42776 | CG18419 | | | CG33298 | lethal | |  |
| 27528 | CG5844 | | | CG5844 | lethal | |  |
| 26075 | CG4599 | | | Tpr2 | lethal | |  |
| 14268 | CG4086 | | | Su(P) | lethal | |  |
| 1414 | CG5677 | | | Spase22-23 | lethal | |  |
| 50221 | CG2076 | | | CG2076 | lethal | |  |
| 23556 | CG17935 | | | Mst84Dd | lethal | |  |
| 33516 | CG2272 | | | slpr | lethal | |  |
| 27110 | CG32179 | | | Krn | lethal | |  |
| 27002 | CG9100 | | | Rab30 | lethal | |  |
| 33423 | CG1911 | | | CAP-D2 | lethal | |  |
| 30066 | CG7398 | | | CG8219 | lethal | |  |
| 33507 | CG2253 | | | Upf2 | lethal | |  |
| 8254 | CG7026 | | | CG7026 | lethal | |  |
| **3016** | **CG4001 §** | | | **Pfk** | **lethal** | |  |
| 3166 | CG10778 | | | CG10778 | lethal | |  |
| **3046** | **CG11282 §** | | | **caps** | **lethal** | |  |
| 33523 | CG2321 | | | CG2321 | lethal | |  |
| 39937 | CG17083 | | | CG17083 | lethal | |  |
| 39976 | CG1542 | | | CG1542 | lethal | |  |
| 29295 | CG9836 | | | CG9836 | lethal | |  |
| 48153 | CG15804 | | | Dhc62B | lethal | |  |
| 6098 | CG18549 | | | CG18549 | lethal | |  |
| 4801 | CG15744 | | | CG15744 | lethal | |  |
| 43944 | CG1965 | | | CG1965 | lethal | |  |
| 26277 | CG3733 | | | Chd1 | lethal | |  |
| **42485** | **CG3297 §** | | | **mnd** | **lethal** | |  |
| 19616 | CG15816 | | | NA | lethal | |  |
| 27607 | CG4364 | | | CG4364 | lethal | |  |
| 35162 | CG7034 | | | sec15 | lethal | |  |
| 15736 | CG6443 | | | CG6443 | lethal | |  |
| 32085 | CG14034 | | | NA | lethal | |  |
| **28982** | **CG8887 §** | | | **ash1** | **lethal** | |  |
| 40013 | CG15666 | | | CG15666 | lethal | |  |
| 40278 | CG31551 | | | CG31551 | lethal | |  |
| 40218 | CG31256 | | | Brf | lethal | |  |
| 21937 | CG4738 | | | Nup160 | lethal | |  |
| 37329 | CG31522 | | | CG31522 | lethal | |  |
| 27680 | CG6603 §^n.a^. | | | Hsc70Cb | lethal | |  |
| 47731 | CG5676 | | | CG5676 | lethal | |  |
| 31364 | CG10837 | | | eIF-4B | lethal | |  |
| 7965 | CG8975 | | | RnrS | lethal | |  |
| **37699** | **CG5748 §** | | | **Hsf** | **lethal** | |  |
| 22019 | CG5160 | | | CG5160 | lethal | |  |
| 41917 | CG3339 | | | CG3339 | lethal | |  |
| 16334 | CG11870 | | | CG11870 | lethal | |  |
| 24992 | CG2779 | | | Muc11A | lethal | |  |
| 2912 | CG13425 | | | bl | lethal | |  |
| 40932 | CG6475 | | | CG6475 | lethal | |  |
| 26325 | CG32202 | | | CG32202 | lethal | |  |
| 29337 | CG9938 | | | Ndc80 | lethal | |  |
| 34737 | CG3923 §^n.a^. | | | Exp6 | lethal | |  |
| 27486 | CG5692 | | | raps | lethal | |  |
| 34331 | CG5596 | | | Mlc1 | lethal | |  |
| 17463 | CG14286 | | | CG14286 | lethal | |  |
| 21563 | CG5323 | | | CG5323 | lethal | |  |
| 35343 | CG8108 | | | CG8108 | lethal | |  |
| 29070 | CG9177 | | | eIF5 | lethal | |  |
| **39529** | **CG17743 §** | | | **pho** | **lethal** | |  |
| 36584 | CG9973 | | | CG9973 | lethal | |  |
| 11210 | CG9633 | | | RpA-70 | lethal | |  |
| 28895 | CG8351 | | | Tcp-1eta | lethal | |  |
| 26275 | CG3714 | | | CG3714 | lethal | |  |
| 26227 | CG3542 | | | CG3542 | lethal | |  |
| 49168 | CG14210 | | | CG14210 | lethal | |  |
| 11205 | CG12005 | | | Mms19 | lethal | |  |
| 21845 | CG4389 | | | CG4389 | lethal | |  |
| 27943 | CG7293 | | | Klp68D | lethal | |  |
| 16091 | CG10920 | | | CG10920 | lethal | |  |
| 3909 | CG10165 | | | CG10165 | lethal | |  |
| **21782** | **CG4062 §** | | | **Aats-val** | **lethal** | |  |
| 20144 | CG12085 | | | pUf68 | lethal | |  |
| 11227 | CG6349 | | | DNApol-alpha180 | lethal | |  |
| 12920 | CG7929 | | | ocn | lethal | |  |
| 13566 | CG7665 | | | Fsh | lethal | |  |
| 12662 | CG6545 | | | lbe | lethal | |  |
| 36308 | CG5528 | | | Toll-9 | lethal | |  |
| 33650 | CG7686 | | | CG7686 | lethal | |  |
| 28396 | CG5684 | | | Pop2 | lethal | |  |
| 41740 | CG30390 | | | Sgf29 | lethal | |  |
| 41964 | CG3820 §^n.a^. | | | Nup214 | lethal | |  |
| 32025 | CG12812 | | | Fancl | lethal | |  |
| **6236** | **CG1378 §** | | | **tll** | **lethal** | |  |
| 7563 | CG14077 | | | CG14077 | lethal | |  |
| 51496 | CG13077 | | | CG13077 | lethal | |  |
| 26309 | CG3931 | | | Rrp4 | lethal | |  |
| 5150 | CG5950 | | | SrpRbeta | lethal | |  |
| 18762 | CG12630 | | | tio | lethal | |  |
| 51846 | CG1571 | | | CG1571 | lethal | |  |
| 29072 | CG9198 | | | shtd | lethal | |  |
| 40834 | CG13431 | | | Mgat1 | lethal | |  |
| 27831 | CG7067 | | | NitFhit | lethal | |  |
| 42976 | CG12050 | | | CG12050 | lethal | |  |
| 30442 | CG4079 | | | Taf11 | lethal | |  |
| 35452 | CG9480 | | | Glycogenin | lethal | |  |
| 23028 | CG8086 | | | CG8086 | lethal | |  |
| 27598 | CG6369 | | | Smg6 | lethal | |  |
| 27600 | CG6375 | | | pit | lethal | |  |
| **38637** | **CG12238 §** | | | **e(y)3** | **lethal** | |  |
| 16744 | CG15749 | | | dmrt11E | lethal | |  |
| 27515 | CG5788 | | | UbcD10 | lethal | |  |
| 41819 | CG3358 | | | CG3358 | lethal | |  |
| **28058** | **CG7516 §** | | | **l(2)34Fd** | **lethal** | |  |
| 35611 | CG7052 | | | TepII | lethal | |  |
| 41885 | CG32376 | | | CG32376 | lethal | |  |
| 34070 | CG5374 | | | T-cp1 | lethal | |  |
| 27457 | CG5546 | | | MED19 | lethal | |  |
| 49345 | CG9155 | | | Myo61F | lethal | |  |
| 48835 | CG7281 | | | CycC | lethal | |  |
| 48793 | CG33051 | | | CG33051 | lethal | |  |
| 48708 | CG10315 | | | eIF2B-delta | lethal | |  |
| 35222 | CG7376 | | | CG7376 | lethal | |  |
| **27870** | **CG7128 §** | | | **Taf8** | **lethal** | |  |
| **31320** | **CG10687 §** | | | **Aats-asn** | **lethal** | |  |
| 31311 | CG10645 | | | lama | lethal | |  |
| **31333** | **CG10719 §** | | | **brat** | **lethal** | |  |
| 35107 | CG6620 | | | ial | lethal | |  |
| 6543 | CG7398 | | | Trn | lethal | |  |
| 14444 | CG6343 | | | ND42 | lethal | |  |
| 26585 | CG4649 | | | Sodh-2 | lethal | |  |
| 41351 | CG31015 | | | PH4alphaPV | lethal | |  |
| 40727 | CG9004 | | | CG9004 | lethal | |  |
| 30673 | | CG6772 | Slob | | | lethal |  |
| 44146 | | CG12752 | Nxt1 | | | lethal |  |
| 8784 | | CG10808 | synaptogyrin | | | lethal |  |
| 17701 | | CG13778 | Mnn1 | | | lethal |  |
| 29462 | | CG7564 | CG7564 | | | lethal |  |
| 40665 | | CG9705 | CG9705 | | | lethal |  |
| 14107 | | CG32374 | CG32374 | | | lethal |  |
| 30140 | | CG6249 | Csl4 | | | lethal |  |
| 36085 | | CG9049 | hiw | | | lethal |  |
| 36086 | | CG9124 | eIF-3p40 | | | lethal |  |
| 32749 | | CG16837 | CG16837 | | | lethal |  |
| 36092 | | CG9200 | Atac1 | | | lethal |  |
| 35165 | | CG7070 | PyK | | | lethal |  |
| 46903 | | CG14230 | CG14230 | | | lethal |  |
| 49030 | | CG5440 | CG5440 | | | lethal |  |
| 32612 | | CG15481 | Ski6 | | | lethal |  |
| 51088 | | CG4602 | Srp54 | | | lethal |  |
| 34388 | | CG7861 | tbce | | | lethal |  |
| 22108 | | CG5383 | PSR | | | lethal |  |
| 51202 | | CG9452 | CG9452 | | | lethal |  |
| 31206 | | CG10230 | Rpn9 | | | lethal |  |
| 15872 | | CG3069 | Taf10b | | | lethal |  |
| 8573 | | CG18578 | Ugt86Da | | | lethal |  |
| 22773 | | CG11985 | CG11985 | | | lethal |  |
| 31216 | | CG10308 | CycJ | | | lethal |  |
| 20567 | | CG1789 | CG1789 | | | lethal |  |
| 15791 | | CG8695 | LvpL | | | lethal |  |
| 41703 | | CG2854 | CG2854 | | | lethal |  |
| 50126 | | CG10098 | CG10098 | | | lethal |  |
| 36205 | | CG9995 | htt | | | lethal |  |
| 39920 | | CG30144 | CG33786 | | | lethal |  |
| 34847 | | CG4448 | wda | | | lethal |  |
| 24054 | | CG9527 | CG9527 | | | lethal |  |
| 50176 | | CG3022 | GABA-B-R3 | | | lethal |  |
| 32521 | | CG31809 | CG31809 | | | lethal |  |
| **52549** | | **CG9191 §** | **Klp61F** | | | **lethal** |  |
| 4659 | | CG8657 | Dgkepsilon | | | lethal |  |
| 23037 | | CG8107 | CalpB | | | lethal |  |
| 12618 | | CG6258 | RfC38 | | | lethal |  |
| 6053 | | CG14709 | CG14709 | | | lethal |  |
| 50021 | | CG11276 | RpS4 | | | lethal |  |
| 40497 | | CG4005 | yki | | | lethal |  |
| 28172 | | CG7989 | wcd | | | lethal |  |
| 6832 | | CG4907 | CG4907 | | | lethal |  |
| 25967 | | CG4247 | mRpS10 | | | lethal |  |
| 24835 | | CG1404 | ran | | | lethal |  |
| 36428 | | CG5742 | CG5742 | | | lethal |  |
| 5985 | | CG14690 | tomboy20 | | | lethal |  |
| 49325 | | CG32708 | CG32708 | | | lethal |  |
| 31271 | | CG10578 | DnaJ-1 | | | lethal |  |
| 46584 | | CG9899 | CG9899 | | | lethal |  |
| 16313 | | CG11804 | ced-6 | | | lethal |  |
| 16806 | | CG16804 | spt4 | | | lethal |  |
| 34498 | | CG3229 | CG33123 | | | lethal |  |
| 12392 | | CG5367 | CG5367 | | | lethal |  |
| 40276 | | CG31531 | CG31531 | | | lethal |  |
| 36440 | | CG5021 | CG5021 | | | lethal |  |
| 39134 | | CG31155 | Rpb7 | | | lethal |  |
| 34395 | | CG7860 | CG7860 | | | lethal |  |
| 16696 | | CG11395 | CG11395 | | | lethal |  |
| **22123** | | **CG5429 §** | **Atg6** | | | **lethal** |  |
| 26299 | | CG10797 | dnc | | | lethal |  |
| 51472 | | CG6582 | Aac11 | | | lethal |  |
| 36557 | | CG6701 | CG6701 | | | lethal |  |
| 17946 | | CG12959 | CG34365 | | | lethal |  |
| 14900 | | CG1627 | NA | | | lethal |  |
| 47248 | | CG15776 | CG42265 | | | lethal |  |
| 49950 | | CG17835 | inv | | | lethal |  |
| 46191 | | CG9288 | CG9288 | | | lethal |  |
| **27503** | | **CG5753 §** | **stau** | | | **lethal** |  |
| 33705 | | CG30327 | CG42257 | | | lethal |  |
| 30537 | | CG11641 | pdm3 | | | lethal |  |
| 5094 | | CG12139 | Megalin | | | lethal |  |
| 31318 | | CG10662 | sick | | | lethal |  |
| 51705 | | CG11105 | CG42683 | | | lethal |  |
| 16331 | | CG11861 | Cul-3 | | | lethal |  |
| 32482 | | CG2578 | Ten-a | | | lethal |  |
| 28628 | | CG7826 | mnb | | | lethal |  |
| 12616 | | CG9207 | Gas41 | | | lethal |  |
