# Supplementary material for: Large-Scale Screen for Modifiers of Ataxin-3-Derived Polyglutamine-Induced Toxicity in Drosophila
Source: PLoS One. 2012 Nov 5;7(11):e47452. doi: 10.1371/journal.pone.0047452 (PMC3489908; doi:10.1371/journal.pone.0047452)
Supplement: Table S2 — Rescue of lethality induced by pan-neural polyQ expression. Table lists transformant ID (from VDRC), gene ID and gene name (if applicable) of all RNAi lines (genes silenced) which were tested for rescue effects on elav>polyQ-induced lethality. In the F1 generation (elav>polyQ in combination with respective RNAi line), effects of gene silencing were categorized as rescue (R) if vital offspring was observed, or lethal (L) if no vital offspring was present. Control (white RNAi) is marked in grey. Lines not available for rescue experiments are marked as not analyzed (n.a.). (DOC) [file pone.0047452.s004.doc]

**Supplementary Table S2: Rescue of lethality induced by pan-neural polyQ expression.**

| **Transformant ID** | **CG number** | **Gene name** | **Rescue of *elav>polyQ*-induced lethality** |
| --- | --- | --- | --- |
| 30033 | *CG2759* | *white* | L |
| 8780 | *CG17048* | *CG17048* | R |
| 7903 | *CG9501* | *ppk14* | R |
| 23121 | *CG7123* | *LanB1* | R |
| 44362 | *CG9131* | *slmo* | R |
| 37221 | *CG9153* | *CG9153* | R |
| 15789 | *CG8696* | *LvpH* | R |
| 26465 | *CG4264* | *Hsc70-4* | R |
| 50222 | *CG4264* | *Hsc70-4* | R |
| 11219 | *CG3284* | *RpII15* | R |
| 3780 | *CG5799* | *dve-s* | R |
| 24030 | *CG9448* | *trbd* | R |
| 40006 | *CG15618* | *CG15618* | R |
| 41530 | *CG14514* | *Brd8* | R |
| 23637 | *CG8863* | *Droj2* | n.a. |
| 43870 | *CG7108* | *DNApol-alpha50* | n.a. |
| 45596 | *CG8937* | *Hsc70-1* | R |
| 41696 | *CG2720* | *Hop* | R |
| 40044 | *CG16890* | *CG16890* | R |
| 8070 | *CG13969* | *bwa* | R |
| 6282 | *CG6755* | *EloA* | R |
| 31257 | *CG10545* | *Gbeta13F* | R |
| 19066 | *CG1658* | *Doa* | R |
| 33262 | *CG5687* | *CG5687* | R |
| 13005 | *CG9695* | *Dab* | n.a. |
| 16182 | *CG1107* | *aux* | R |
| 37930 | *CG14619* | *CG14619* | R |
| 46473 | *CG17919* | *CG17919* | n.a. |
| 43606 | *CG6758* | *CG6758* | R |
| 43802 | *CG6363* | *MRG15* | n.a. |
| 8408 | *CG3389* | *Cad88C* | R |
| 34713 | *CG3808* | *CG3808* | R |
| 25030 | *CG31110* | *5PtaseI* | R |
| 26475 | *CG4266* | *CG4266* | R |
| 23843 | *CG16807* | *roq* | n.a. |
| 16040 | *CG10377* | *Hrb27C* | R |
